# Effects of a Buried Cysteine-To-Serine Mutation on Yeast Triosephosphate Isomerase Structure and Stability

**DOI:** 10.3390/ijms130810010

**Published:** 2012-08-10

**Authors:** Alejandra Hernández-Santoyo, Lenin Domínguez-Ramírez, César A. Reyes-López, Edith González-Mondragón, Andrés Hernández-Arana, Adela Rodríguez-Romero

**Affiliations:** 1 Instituto de Química, Universidad Nacional Autónoma de México, Circuito Exterior, CU México D.F. 04510, Mexico; E-Mail: hersan@unam.mx; 2 División de Ciencias Biológicas y de la Salud, Universidad Autónoma Metropolitana, Lerma, Lerma de Villada 07360, Mexico; E-Mail: j.dominguez@correo.ler.uam.mx; 3 Laboratorio de Investigación Bioquímica, Postgrado Institucional en Biomedicina Molecular, ENMyH-Instituto Politécnico Nacional, CP 07320 México, DF, Mexico; E-Mail: careyes@ipn.mx; 4 Instituto de Agroindustrias, Universidad Tecnológica de la Mixteca, Huajuapan de León, Oaxaca 69000, Mexico; E-Mail: edithgonmon@yahoo.com.mx; 5 Departamento de Química, Universidad Autónoma Metropolitana-Iztapalapa, Iztapalapa 09340, D.F., Mexico

**Keywords:** crystal structure, molecular dynamics, *Saccharomyces cerevisiae*, stability, triosephosphate isomerase

## Abstract

All the members of the triosephosphate isomerase (TIM) family possess a cystein residue (Cys126) located near the catalytically essential Glu165. The evolutionarily conserved Cys126, however, does not seem to play a significant role in the catalytic activity. On the other hand, substitution of this residue by other amino acid residues destabilizes the dimeric enzyme, especially when Cys is replaced by Ser. In trying to assess the origin of this destabilization we have determined the crystal structure of *Saccharomyces cerevisiae* TIM (ScTIM) at 1.86 Å resolution in the presence of PGA, which is only bound to one subunit. Comparisons of the wild type and mutant structures reveal that a change in the orientation of the Ser hydroxyl group, with respect to the Cys sulfhydryl group, leads to penetration of water molecules and apparent destabilization of residues 132–138. The latter results were confirmed by means of Molecular Dynamics, which showed that this region, in the mutated enzyme, collapses at about 70 ns.

## 1. Introduction

Due to the chemical reactivity of the thiol group, cysteine residues are frequently found as constituents of the catalytic machinery of enzymes, as ligands of metallic ions or more recently as glutathionylated residues. In those particular cases, stringent conservation of the Cys residue is observed in sequence alignments of different proteins with similar biological activity. Nevertheless, examples are known of buried Cys residues that, although not playing a significant role in catalysis or metal binding, have remained invariant through evolution [1,2] because they seem to be necessary for efficient folding or structural stability of the protein molecule.

In the family of triosephosphate isomerases (TIM) there is a strictly conserved cysteine, which is located near the end of the β-strand 5, forming part of the hydrophobic core of the (β/α)_8_ barrel-structure displayed by these enzymes [3,4]. The sulfur atom of this invariant cysteine (Cys126, according to the sequence numbering of *Saccharomyces cerevisiae* TIM, ScTIM) is located at van der Waals distance from one of the oxygen atoms of the catalytic Glu165; yet, substitution of Cys126 by Ala or Ser only slightly affects the activity of ScTIM [1]. Mutation of Cys126 has, on the other hand, sound effects on the stability of the native yeast TIM dimer. For instance, the difference in unfolding free energy (ΔΔ*G* = Δ*G*^2U–N^_mutant_ – Δ*G*^2U–N^_wild-type_) for mutants C126A and C126V has been estimated as −15 and −18 kJ mol^−1^, respectively, whereas the destabilization of mutant C126S doubles that value (ΔΔ*G* = −37 kJ mol^−1^) [5]. As several pieces of evidence indicate, the temperature-induced unfolding of ScTIM conforms with a two-state process [5,6]. Therefore, the unfolding free energy can be calculated as follows: Δ*G*^2U–N^ = −R*T*ln *K*_u_ = −R*T*ln (*k*_u_/*k*_r_), where *k*_u_ and *k*_r_ represent the kinetic rate-constants for unfolding and refolding, respectively. It has been found that the main factor contributing to the strikingly high ΔΔ*G* for the C126S enzyme is its very large rate constant for unfolding (100- to 1000-fold larger than for the C126V and C126A mutants, at 37 °C). A smaller contribution to ΔΔ*G* comes from the rate constant for refolding, which for the C126S variant is about 10-fold smaller than for the other two mutants [5].

In a previous study [1], a hypothesis was advanced to explain the large destabilization of ScTIM C126S. By using well-known correlations between the energetics of protein stability and solvent-accessible surface areas, it was proposed that the native state in the C126S mutant is more hydrated than the native state of wild-type TIM. Recently, it has been reported that a number of *Plasmodium falciparum* TIM (PfTIM) mutants of Cys126 are notably less stable than the wild type enzyme [7]. Even though Cys126 participates in no direct monomer-monomer contacts, Samanta *et al*. [7] suggest that its substitution (by Ala or Ser) results in the perturbation of interactions involving residues from both subunits, thus leading to destabilization of the native dimer.

To find out what structural modifications occur in the molecule of yeast TIM when Ser replaces Cys126, we determined the crystal structure of the C126S mutant at 1.86 Å resolution. The structure showed the presence of the substrate analog 2-phosphoglycolate (PGA) in monomer A only. When compared to the structure of the wild-type ScTIM (PDB code 1YPI, [3]), the mutant C126S revealed relatively minor alterations in the local environment of residue 126, as in the case of the *Plasmodium. falciparum* enzyme; of the changes observed, the most important is the formation of a hydrogen bond between OE1-Glu165 and the OG of the newly introduced Ser residue. This local change, however, seems to affect the network of water molecules extending from the central β-barrel to the molecular surface.

## 2. Results and Discussion

### 2.1. Enzyme Preparation, and Crystallization

After expression in *Escherichia coli*, sequential purification using size-exclusion and anionic-exchange chromatography yielded a homogeneous preparation of the C126S ScTIM mutant. Determinations of enzymatic activity showed that *k*_cat_ and *K*_M_ of the mutant enzyme were both reduced about four fold with respect to those of wild-type ScTIM. As stated previously, however, these minor, compensatory changes in k_cat_ and K_M_ result in a catalytic efficiency (*k*_cat_/*K*_M_) value that is only subtly affected [1].

Attempts to crystallize the mutant without PGA were unsuccessful; nonetheless, crystals in the presence of PGA grew in about 3 weeks with a rod-shaped morphology and diffracted to 1.86 Å resolution. The analysis of the diffraction pattern showed that they belonged to the space group P2_1_2_1_2_1_ with unit cell parameters (Å) of a = 46.92, b = 61.44 and c = 160.24. The calculated Matthews’ coefficient [8] for two monomers per asymmetric unit is 2.31 Å and gives an estimated solvent content of 46.8%. The dimer interface buries a surface of 1642 Å^2^ per monomer compared to 1603 Å^2^ for 1YPI. The refined structure contains two monomers, each consisting of 247 amino acid residues and 318 ordered water molecules, with a final *R*_work_ and *R*_free_ values of 17.5 and 21.2, respectively. Details of the X-ray data collection and structure refinement are listed in Table 1.

### 2.2. Crystal Structure Overview

**Figure 1:**
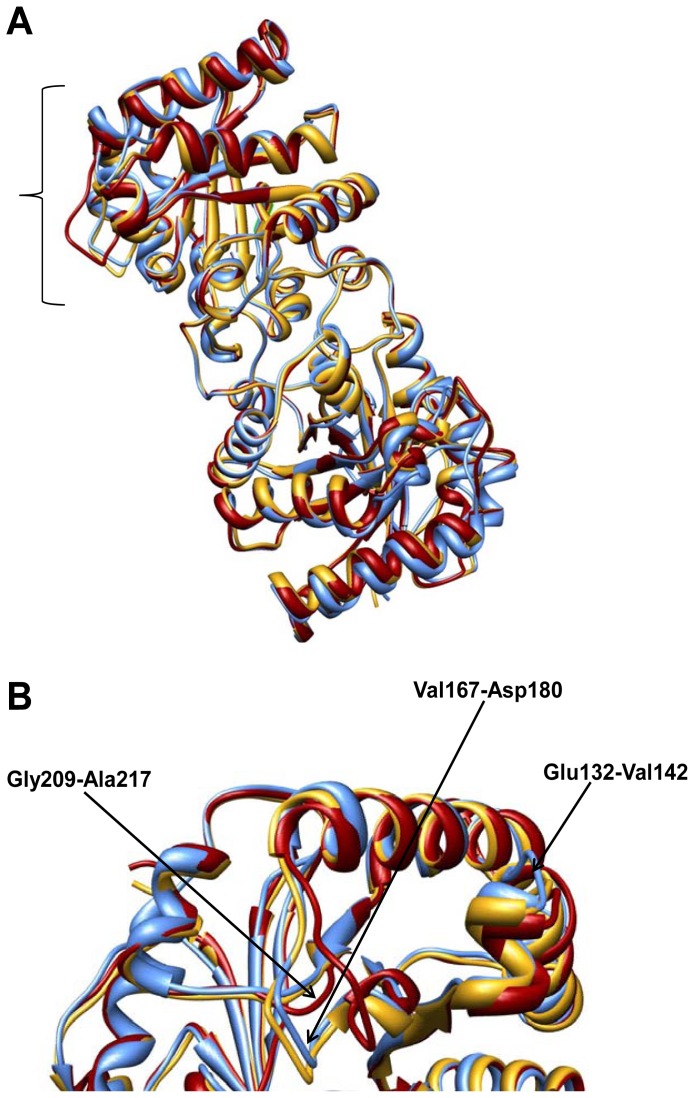
Structural alignment of triosephosphate isomerases from *Saccharomyces. cerevisiae*. (**A**) Mutant C126S (gold) and the wild-type enzyme in the absence (red, 1YPI) and in the presence of PGA (blue, 2YPI); (**B**) Close-up view of the three regions with major differences: Glu132-Val142, Val167-Asp180 and Gly209-Ala217.

The structure of C126S ScTIM shows the canonical features conserved for this protein, with the common (β/α)_8_ TIM-barrel fold consisting of a barrel of eight β-strands surrounded by helices. Overviews of the C_α_ superposition of the mutant and the wild type enzymes, in the absence and presence of PGA [PDB codes 1YPI and 2YPI] are shown in Figure 1A. The C_α_ RMSD values for the dimers are 0.44 Å, and 0.81 Å, respectively, while the RMSD value for both monomers in the mutant is 0.40 Å, indicating that a very high structural similarity exists. Nonetheless, there are three regions (Glu132-Val142, Val167-Asp180 and Gly209-Ala217) that depict different conformations, as shown in Figure 1B. The most important difference is observed from residues Val167 to Asp180 that correspond to loop 6, which is open in the monomer without PGA. An analysis of the CA superpositions for the wild-type (PDB codes 1YDV and 2VFI) and mutated PfTIMs [PDB codes 3PY2 and 3PVF] demonstrated that these structures are very similar with Cα RMSD values ranging from 0.3 to 0.52 Å and showed two regions with important differences in conformation (residues 167–180 and residues 209–217) (Supplementary Figure S1). In this case the region comprising residues 132–142 is very similar in the mutated and the wild type enzymes.

Regardless of the similar structures, the electron densities at the active sites of the C126S ScTIM monomers were considerably different. The electron density observed at the active site of monomer B did not account for an intact PGA; therefore, we interpreted this density as a sulfate ion and glycerol with occupancies of 1.0. It is important to mention that the catalytic loop or loop 6 presents a “closed” conformation in monomer A (with PGA), and the “open” conformation in monomer B, which contains glycerol and a sulfate ion, a situation that has been observed before for *P. falciparum* TIM in the presence of 2-phosphoglycerate (2PG) [9]. The authors explained that due to radiation damage a broken inhibitor was found in one subunit and its catalytic loop presented an “open” conformation, while the other subunit presented alternative conformations. In our structure, PGA is bound to the catalytic site in monomer A with a tight hydrogen-bond network including NZ-Lys12, NE2-His95, OE2-Glu165, N-Gly171, N-Ser211, N-Gly232 and N-Gly233, which are structurally conserved among different structures.

**Figure 2:**
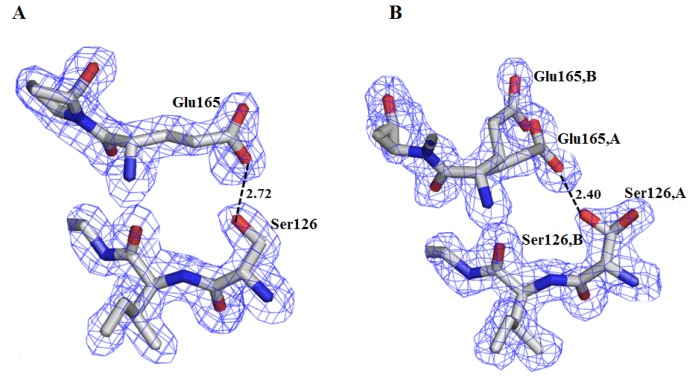
2Fo-Fc electron density map contoured at 1.0 σ showing the mutation (Ser126) and the new hydrogen bond established with Glu165. (**A**) Monomer A (ligand-bound); (**B**) Monomer B (ligand-free). In the latter, both Ser126 and Glu165 present a double conformation.

Figure 2A,B shows 2Fo-Fc electron density maps contoured at 1.0 σ around the mutation, where it is observed that Ser126 in the ligand-bound monomer presents one conformation, whereas in the ligand-free monomer this residue and Glu165 adopt double conformations. In both monomers OG-Ser126 establishes a hydrogen bond with OE1-Glu165. In contrast, in the wild-type structure the SG of Cys126 is oriented towards the interior of the β-barrel and it does not interact with Glu165.

**Figure 3:**
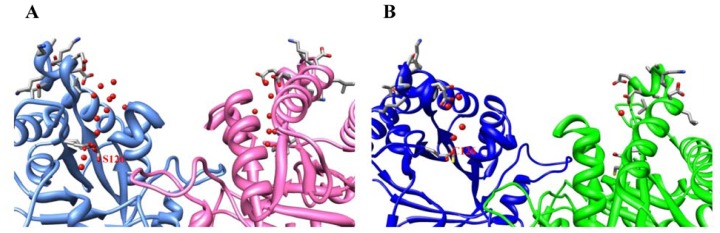

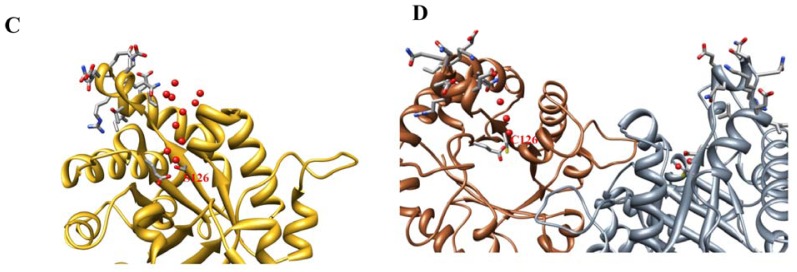
Waters surrounding residue 126 in ScTIM and PfTIM. (**A**) ScTIM C126S mutant; (**B**) wild-type ScTIM (PDB code 1YPI; (**C**) PfTIM C126S mutant (PDB code 3PVF and (**D**) wild-type PfTIM (PDB code 2VFI). It is also shown the side chains of residues 132–142, which are close to the mutation site and present slightly different conformation between the wild-type and C126S ScTIMs. In A, B and D monomer B is at the left side of the figure.

The main local changes around the mutation include the presence of several water molecules, not observed in the wild type enzyme (Figure 3A,B, respectively). In monomer A two water molecules establish three hydrogen bonds with Ser126, whereas in monomer B three water molecules form four hydrogen bonds with this residue. In contrast, Cys126 in the wild-type protein interacts with a single water molecule (Figure 3B). It is interesting to note that the recently reported structure of PfTIM C126S mutant also shows more waters around the mutation than the wild type enzyme (PDB codes 3PVF and 2VFI, respectively), as observed in Figure 3C,D. A detailed comparison of the C126S mutants in ScTIM and PfTIM is shown in Supplementary Figure S2. This superposition shows that the site of mutation and the region comprising residues 132–142 are very similar.

Another interesting characteristic of the C126S ScTIM mutant is that the accessibility of the polar and non-polar residues is different. Considering the residues which are 5% solvent exposed, in both 1YPI and the mutant, as determined in NACCESS (S.J. Hubbard, J.M. Thornton, University College, London, 1993), it is observed that 296 non-polar residues and 278 polar residues are exposed to the solvent in the former, while 293 non-polar and 260 polar are exposed in the latter. Residues having less than 5% of their surface accessible to the solvent are considered buried, as described by Miller *et al.* [10].

Global surface area calculations, however, resulted in only small differences between the C126S mutant and the wild-type molecules; if the ligand-free monomers are compared, the mutant exposes to the solvent about 100 Å^2^ more of non-polar area, but ca. 150 Å^2^ less of polar area, than the wild-type protein. These differences in solvent-accessible surface area represent, nevertheless, only 1%–2% of the total area exposed by the monomer. Similar calculations carried out with the PfTIM structures indicated that the non-polar area exposed is 2% less in the C126S mutant than in the wild-type molecules; the polar area is only 0.2% smaller in the mutant. For the ScTIM enzymes, the minor differences in solvent-exposed surface area mentioned above seem to disagree with the previous finding that the C126S mutant displays a ΔCp for refolding less negative than the wild-type enzyme [1]. Indeed, the observed difference in ΔCp seems to indicate that the native C126S mutant buries a few thousand square ångströms less than the wild-type molecules. This difference in buried area, however, should be regarded only in qualitative terms due to the uncertainty introduced by the relatively large experimental error involved in the ΔCp determination. It is evident that the molecular origin of the observed differences in ΔCp must be sought in other structural properties, different from the total accessible surface area. In this regard, one likely explanation for the above mentioned discrepancies might be the more extensive hydration observed in the internal region of the C126S molecule that is close to the site of mutation. That is, it may be thought that in the presence of a large number of water molecules, an internal region of the protein can change its *buried*, mainly apolar, character to a more polar one.

### 2.3. Molecular Dynamics

**Figure 4:**
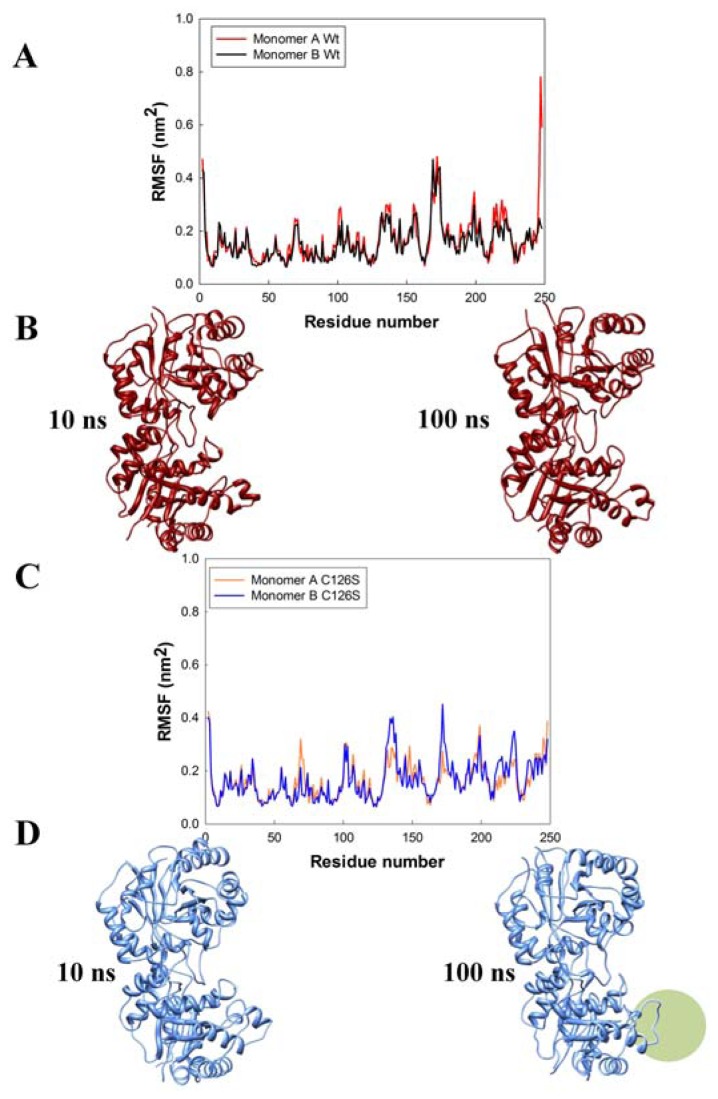
Root mean square fluctuations (RMSF) of the backbone atoms during molecular dynamics simulations of ScTIM (**A**) and the C12S mutant (**C**); Panels (**B**) and (**D**) show secondary structural elements present at 10 and 100 ns of molecular dynamics for the wild type ScTIM and the mutant, respectively. The 132–142 region that collapses at 100 ns is labeled with a green circle.

In conjunction with the structural analysis, MD simulations evaluated the effect of the Sc-C126S mutation and the results were compared with those obtained for the wild-type structure 1YPI. Root Mean Square Fluctuations (RMSF) over the whole simulation period of 100 ns for the monomers of the wild type and the mutated ScTIM are shown in Figure 4. It is clearly evident that some regions have large atomic mobilities, along the dynamics trajectory. A high RMSF is observed in the catalytic loop (residues ~166–181) for both proteins, whereas in monomer B of the C126S mutant above average fluctuations are also observed in the region formed by residues Glu132-Val142 (Figure 4A,C).

In this regard, snapshots of the 10 and 100 ns MD simulations for the two dimers showing the time evolution of the secondary structure elements (Figure 4B,D) are interesting. The most striking result from molecular dynamics is, perhaps, the structural disorder exhibited by the segment comprising residues Glu132 to Val142 in the C126S monomer B molecule, which collapses at about 100 ns (Figure 4D). As described above, this region of the molecule is located near the site of mutation (Figure 3) and appears to be significantly modified in the TIM structure when Cys126 is substituted by Ser, probably as a result of the rearrangement of hydrogen bonds that occurs due to the presence of numerous water molecules. Even though the results from molecular dynamics computations were obtained at only one temperature (300 K), they point to a possible molecular origin of the large unfolding rate of the C126S mutant dimer. For instance, it has been determined that at 37 °C (317 K) the unfolding-kinetics constant for this mutant is approximately four orders of magnitude larger than that for wild-type TIM [5]. Thus it is interesting to perform further simulations at higher temperatures and in the presence of ligands.

## 3. Experimental Section

### 3.1. Protein Expression, Purification and Crystallization

The recombinant C126S variant of *S. cerevisiase* TIM was expressed and purified as described previously [1]. Briefly, the mutated gene was cloned in the pET3a vector (pET System, Novagen); codon substitutions were confirmed by sequencing. The resulting plasmid was used to transform *E. coli* BL21 (DE3) pLysS cells. Over-expression and purification of C126S TIM was carried out as described by Vázquez-Contreras *et al*. [11] using a two-step purification procedure until the mutant was found to be homogeneous, as determined by SDS-PAGE and anion-exchange FPLC analysis. Protein concentration was determined from the absorbance at 280 nm, using the absorption coefficient (A^1%^ =10) [12]. Enzymatic activity was assayed at 25 °C, as described before [1,13], using d-glyceraldehyde 3-phosphate as the substrate.

For crystallization, the purified C126S ScTIM was dialyzed against 25 mM triethanolamine, 10 mM EDTA, pH 7.4, and concentrated. Crystals were grown at 18 °C, by the vapor diffusion method from hanging drops. Two drops were set in each experiment by mixing 4 μL of C126S mutant (2.3 mg mL^−1^) with 4 μL of reservoir solution (ammonium sulfate 2.2 M, 0.01 M cobaltous chloride hexahydrate in 0.01 M MES pH 6.5), but in one of the drops 1 μL of 4 mM PGA was also added. Crystals were only obtained in the drops containing PGA. Data were collected on a single flash-cooled crystal using 30% glycerol as cryoprotectant at National Synchrotron Light Source (Upton, NY, USA) on a Quantum 210 CCD detector (Area Detector System Corporation, Poway, CA, USA) at beamline X6A. Data were integrated using the program XDS [14] and scaled with SCALA [15,16].

### 3.2. Structural Determination and Refinement

The Sc C126S TIM crystal phases were determined by molecular replacement using the coordinates of ScTIM (PDB ID 1YPI) and the program PHASER [17] (*Z*-score 76.2, LLG 5079.04). The atomic positions obtained were used to initiate phase improvement by torsion non-crystallographic symmetry (NCS) averaging as implemented in PHENIX [18]. The resulting electron density maps were used to build the C126S structure and initiate crystallographic refinement that included rigid body, real space, individual atomic coordinate and individual atom isotropic displacement parameter strategies. Manual model adjustment to improve the fit to likelihood-weighted electron-density maps was carried out using COOT [19]. In the final refinement cycles, NCS restraints were released and addition of PGA, ions and water molecules allowed construction of the final model. The stereochemistry and the agreement between the model and the X-ray data were verified using COOT and MOLPROBITY [20]. Molecular comparisons were performed with ALIGN [21] and the figures were prepared with PyMOL [22] and Chimera [23]. Accessible surface-area calculations and the interfacial areas determination on the dimers were performed using NACCESS (S.J. Hubbard, J.M. Thornton, University College, London, UK, 1993). The distance calculations considered residues within 4.0 Å of the protein molecules.

### 3.3. Molecular Dynamics

For molecular dynamics simulations the suite GROMACS [24] 4.5.4 compiled in its double precision version was used and the GROMOS96 43a1 force field was employed; the time step was 4.0 fs and hydrogen virtual sites were employed; SHAKE was used for all covalent bonds. The short-range forces (Lennard-Jones and Coulomb) were cut off at 1.0 nm. Long-range forces (Coulomb) were cut off at 1.0 nm and updated during generation of the neighbor list every 20 fs. For the simulation of the wild-type enzyme, the PDB ID 1YPI structure was used; non-protein atoms were removed manually before molecular dynamics simulation. Alternative conformation residues were removed from the Sc-TIMC126S structure, leaving those side chains with higher occupancy or lower B-factor. Each dimeric structure was independently solvated in a dodecahedral box with its nearest edge 1.0 nm away from the protein and filled with 21,788 TIP3P water molecules and enough counter ions to achieve an ionic strength 0.1 M. The total number of atoms was 70,155 for 1YPI and 65,793 for C126S ScTIM. Energy minimization was performed using the steepest descent algorithm for 1000 steps. Then, a restrained MD simulation of 200 ps was performed to allow the solvent to relax; the peptide atoms were harmonically restrained to their position in the crystal with a force constant of 1000 kJ per mol per nm^2^. Temperature was controlled by weak coupling to a bath of constant temperature (*T*_0_ = 300 K, coupling time *t*_t_ = 0.1 ps, with a modified Berendsen thermostat), and the pressure was controlled using a weak coupling to a bath at constant temperature (*P*_0_ = 1 bar, coupling time *t*_t_ = 2.0 ps, using Parrinello-Rahman pressure coupling). The free MD run was carried out for 100 nanoseconds with the same pressure- and temperature-coupling constants as the restrained run. All steps of the simulations were performed using periodic boundary conditions.

## 4. Conclusions

The determination and analysis of the structure of the C126S ScTIM mutant has given some clues to explain why this mutant is much more unstable than the wild-type enzyme. The key point seems to be that, due to its highly polar nature, the OH group of the Ser residue turns out from the interior of the β-barrel and, in its new environment, establishes hydrogen bonds with Glu165 and two water molecules. This new rotamer orientation apparently gives rise to penetration of additional water molecules that likely modify the polar/apolar nature of an interior region of the molecule. Furthermore, a long stretch of residues (Glu132-Val142), which becomes unstable, as indicated by MD simulations, may be the origin of the faster unfolding of the C126S mutant with respect to the wild-type Sc-TIM.

## Supplementary Materials



## Figures and Tables

**Table 1:** Data collection and structure refinement statistics.

***Data collection***	

Space group	P2_1_2_1_2_1_
Cell dimensions (Å)	a = 46.92 b = 61.44 c = 160.24 α = β = γ = 90.00
Temperature (K)	100
Wavelength (Å)	0.9791
Resolution limit (Å)	40.49–1.86 (1.93–1.86)
Reflection collected	251,929 (30,945)
Unique reflections a	39,548 (5593)
*R* b_merge_	10.6 (44.3)
Mean *I/σI*	11.4 (3.6)
Completeness (%)	99.7 (98.6)
Redundancy	6.4 (5.5)
Wilson B-factor (Å^2^)	21.45

***Refinement***	

R c_work_/R_free (%)_	17.47 (22.21)/21.20 (25.20)
No. of atoms of protein/solvent	3786/318
No. of residues of	
SO_4_/PO_4_/glycerol/Na/PGA/	2/3/12/1/1
Average B-value (Å^2^)	13.6
Macromolecule	12.7
Solvent	18.1
*R.m.s.d.* from ideal	
Bond lengths (Å)	0.01
Bond angles (°)	1.29
Ramachandran statistics of ϕ/ψ angles (%)	
Most favored	98
Additionally allowed	2
PDB code	4FF7

a Values in parentheses correspond to the last resolution shell;

b R_merge_ = ∑*_j_* ∑*_h_* (|*I_j,h_* − <*I_h_*>|)/∑*_j_* ∑*_h_* (<*I_h_*>), where *h* is the unique reflection index, I*_j,h_* is the intensity of the symmetry-related reflection, and <I*_h_*> is the mean intensity;

c *R* = ∑_h_|| *F*_o_|*_h_* − |*F*_c_|*_h_*|/∑_h_ |*F*_o_|*_h_* for all reflections, where *F*_o_ and F_c_ are observed and calculated structure factors, respectively, and h defines unique reflections. *R*_free_ is calculated analogously for the test reflections, randomly selected, and excluded from the refinement.
