# Supplementary material for: Effects of a Buried Cysteine-To-Serine Mutation on Yeast Triosephosphate Isomerase Structure and Stability
Source: Int J Mol Sci. 2012 Aug 10;13(8):10010–21. doi: 10.3390/ijms130810010 (PMC3431843; doi:10.3390/ijms130810010)
Supplement: Supplementary file 1 [file ijms-13-10010-s001.pdf]

Effects of a Buried Cysteine-To-Serine Mutation on Yeast Triosephosphate Isomerase Structure and Stability

Supplementary Information

Figure S1. Structural superposition of triosephosphate isomerases from *Plasmodium falciparum*. (**A)** Wild-type enzyme without ligand (1YDV, light blue), wild type enzyme with ligand (2VFI, dark blue), C126S mutant with ligand (3PVF, gold) and C126S mutant without ligand (3PY2, green); (**B**) Close-up view of the monomer A that shows the main differences between them.

A

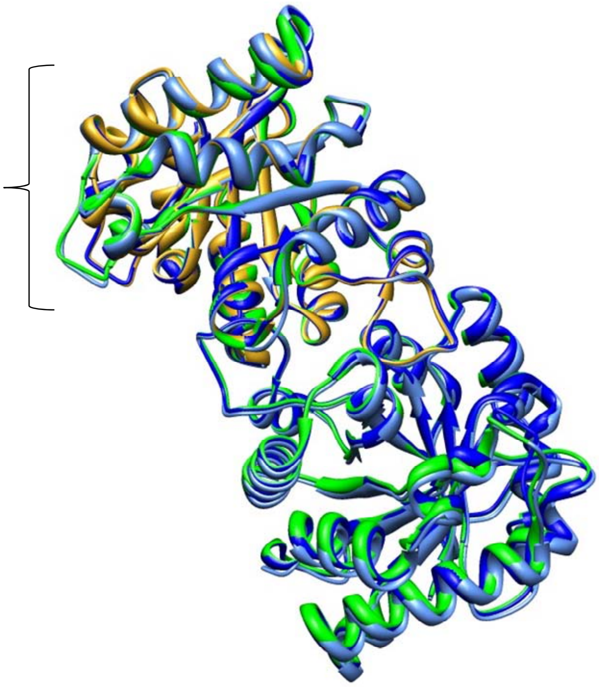

B

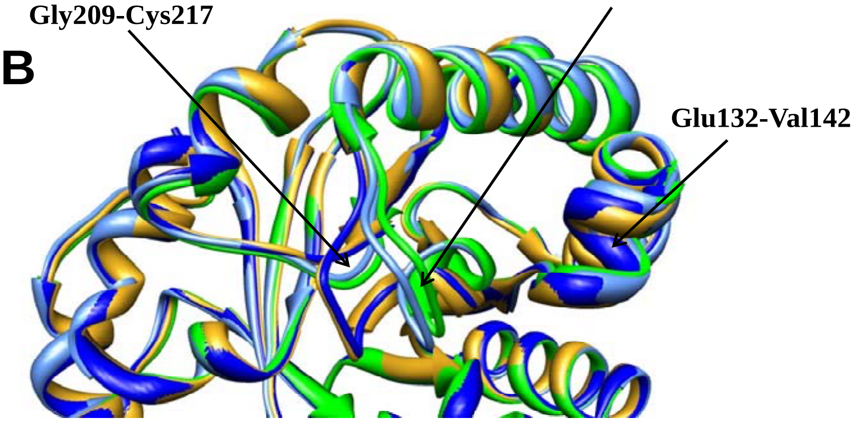

Figure S2. Superposition of the region containing the mutation C126S in the triosephosphate isomerases from *Saccharomyces cerevisiae* (light blue) and *Plasmodium falciparum* (gold). The orientation of Ser126 is the same in both structures. Side chains for residues in the 132–142 region are shown in both structures.

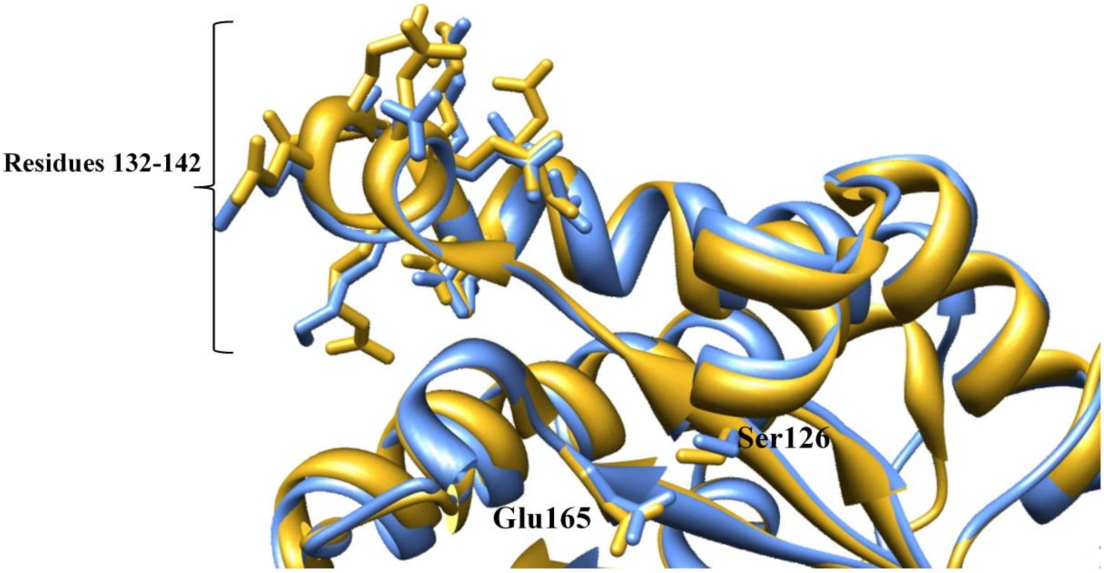

© 2012 by the authors; licensee MDPI, Basel, Switzerland. This article is an open access article distributed under the terms and conditions of the Creative Commons Attribution license (http://creativecommons.org/licenses/by/3.0/).
